# The WHO strategy for prevention and control of snakebite envenoming: a sub-Saharan Africa plan

**DOI:** 10.1590/1678-9199-JVATITD-2019-0083

**Published:** 2019-12-02

**Authors:** Jean-Philippe Chippaux, Achille Massougbodji, Abdulrazaq G. Habib

**Affiliations:** 1 Centre de Recherche d’Île de France, Institut de recherche pour le développement (IRD), Paris, France; 2 Center for Translational Science, Pasteur Institute, Paris, Île-de-France, France; 3 Institut de Recherche Clinique du Bénin (IRCB), Abomey-Calavi, Benin; 4 Department of Medicine, College of Health Sciences, Bayero University Kano (BUK), Kano, Nigeria

**Keywords:** Snakebite, Envenomation, Antivenom, Sub-Saharan Africa, Neglected tropical diseases, Control

## Abstract

Snakebite is a critical public health issue in tropical countries, particularly in Africa, where 20% of snakebites globally occur. In 2017, the WHO added snakebite envenoming to the category A of neglected tropical diseases. In 2019, thanks to broad institutional and international NGO support, including strong mobilization of African experts and governments, WHO launched a strategy for prevention and control of snakebite envenoming with more ambitious goals. In sub-Saharan Africa, accessibility of antivenoms and symptomatic, adjuvant or replacement therapy is a priority. Several antivenoms are available but their evaluation has not been properly carried out and they remain expensive. To date, there are no manufacturers of antivenom in sub-Saharan Africa (except in South Africa), which requires their importation from other continents. The lack of experience in antivenom choice and its use by health authorities, health personnel and population largely explains the shortage in sub-Saharan Africa. The deficiency of epidemiological data does not allow the implementation of appropriate and efficient care. It is crucial to strengthen the health system which does not have the necessary means for emergency management in general and envenoming in particular. Providing peripheral health centers with antivenoms would decrease complications and deaths. The motivation of communities at risk, identified through the epidemiological data, would be to reduce the delay in consultation that is detrimental to the efficiency of treatment. Partnerships need to be coordinated to optimize resources from international institutions, particularly African ones, and share the burden of treatment costs among all stakeholders. We propose here a project of progressive implementation of antivenom manufacturing in sub-Saharan Africa. The various steps, from the supply of appropriate venoms to the production of purified specific antibodies and vial filling, would be financed by international, regional and local funding promoting technology transfer from current manufacturers compensated by interest on the sale of antivenoms.

Snakebite envenoming (SBE) is a critical public health issue in nearly 100 low and middle income tropical countries (LMICs). In sub-Saharan Africa (SSA), there would be nearly 500,000 SBEs annually resulting in about 30,000 deaths and at least as many definitive disabilities [1, 2, 3], which represents more than 20% of all notified SBEs worldwide. These figures are, however, underestimated because of patients’ treatment-seeking behavior that delays access to health centers and increases the risk of death before reaching it. Such a situation results from the high proportion of rural population and the living conditions in SSA, which leads on the one hand to frequent close contact between humans and snakes, and on the other hand to deficient medical care. The population at risk is composed of active people (15-50 years old), mostly male. SBEs occur in rural areas during agricultural and pastoral activities. In LMICS, where more than 99% of SBEs happen, the health facilities and drug supply - particularly antivenoms (AVs) - are defective, which largely explains the high case fatality rates and disappointment of the health staff who lacks means to face such a scourge. The use of traditional medicine is systematic as much to ward off the bad fate - the main cause of accidents according to a majority of the population - as concerning cultural and geographical proximity, and the logistical and financial accessibility of traditional healers [4, 5].

This problem has been pointed out by specialists who have sought to draw the attention of national health authorities and World Health Organization (WHO) for action to be taken. Since the epidemiological report on global snakebites by Swaroop and Grab [6], the WHO has focused on the manufacture and accessibility of AVs. In 1977, the Venom Research Unit established in 1963 by Alistair Reid at the School of Tropical Medicine, Liverpool, was appointed as WHO Collaborating Center for AV Control [7]. Subsequently, the WHO regularly convened experts to discuss the quality of AVs [8, 9, 10, 11, 12]. Until 2010, the main objective of the WHO was to propose recommendations for the manufacture of AVs. In 2017, SBE was added to the category A of neglected tropical diseases (NTDs) [12], and the WHO Snakebite Envenoming Working Group (WHO-SBEWG) was created. The objectives of the WHO-SBEWG were to: strengthen the patient’s management, improve the availability of effective AVs, and reduce morbidity and mortality from SBE. Two years later, following WHO-SBEWG recommendations, WHO launched a strategy for prevention and control of snakebite envenoming [13]. The objectives are more ambitious and a detailed roadmap has been established on a global scale with the following objectives:

- Promote the accessibility of treatments - antivenoms, antidotes, symptomatic, substitutes and adjuvants - combining safety and efficiency.
- Strengthen health systems in an integrative approach.
- Involve, motivate and help communities at risk to take appropriate measures.
- Increase partnerships, coordination and resources through an appropriate global coalition to ensure effective advocacy.

The contributions by WHO have been strongly supported and guided by many experts. For a decade, institutions and non-governmental organizations (NGOs) have also been very active (Global Snakebite Initiative, Health Action International, African Society of Venomology - ASV, Médecins Sans Frontières, International Society of Toxinology in particular). Finally, 18 States mobilized under the initiative of Costa Rica recommended the addition of SBEs to category A of NTDs and the implementation of the strategy of prevention and control (Afghanistan, Angola, Bangladesh, Benin, Burkina Faso, Costa Rica, Cameroon, Chad, Gabon, Guinea, Kenya, Nepal, Nigeria, Pakistan, Papua New Guinea, the Philippines, Senegal and Uganda, totaling 11 countries in SSA, five in Asia, one in the Americas and one in Pacific).

The concerted effort by African countries was largely due to the particular epidemiological situation in SSA, beyond many points in common with those of other parts of the world. The incidence and mortality are very high and management is particularly poor, which leads to an unacceptable burden [2, 14]. In SSA, the objective should be, first and foremost, the improvement of care as recommended by the ASV.

## Treatment Access

The management of SBEs comprises two inseparable components: the AV that should be administered as soon as possible to eliminate the venom from the victim’s body, and the symptomatic, substitute and adjuvant treatments, to stop the evolution of envenoming and relieve the patient. Their effectiveness as well as their safety are crucial, especially in SSA because the rural peripheral health centers that receive most patients lack the necessary means and personnel to treat adverse events.

While there are some AVs available in SSA, the main concerns are related to their poor quality, accessibility and lack of rigorous clinical trials demonstrating their efficacy and safety [15, 16, 17]. However, very few AVs have been appropriately evaluated by robust clinical trials demonstrating both their efficacy and good tolerance [18].

**Figure 1.**
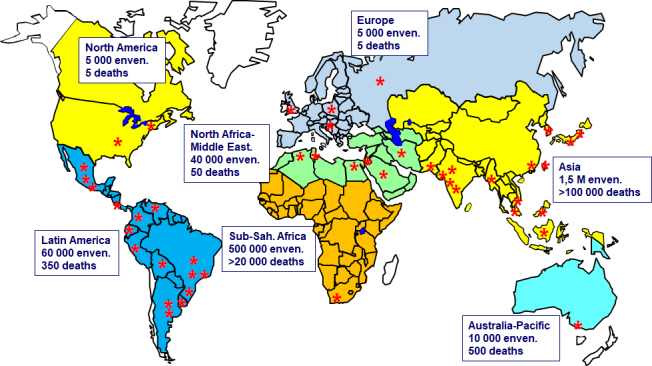
Global distribution of antivenom manufacturers (indicated by red asterisks).

The issue of safety and effectiveness of AVs are similar in all the continents except for SSA, where distribution is particularly precarious, uncertain and expensive. Poor accessibility of AVs has been pointed out twenty years ago [4, 19]. In Asia and Latin America, where there are many manufacturers (Figure 1), the economic model is relatively sound because governments buy a sufficient amount of AVs to treat SBEs occurring in the country, which generally helps to balance manufacturing costs. Local manufacture of AVs, most of which goes back several decades, has thus favored the emergence of regular commercial and therapeutic practices, even though it can be seen that production does not always meet the required quality standards, in terms of both safety and effectiveness. Conversely, in SSA, there is just one AV manufacturer in South Africa that does not have commercial export policy. The SSA - except South Africa and few neighboring countries - is therefore highly dependent on overseas manufacturers, which raises the problem of the validity and availability of the AVs distributed. The current manufacturing standards make the manufacturing of AVs expensive and the market very constrained, which now limits the possibility of developing local manufacturing that many African countries request in order to meet their specific market.

Specific interventions in SSA should be added to the actions advocated by the WHO roadmap to improve the manufacture of AVs and to rationalize the choice and use [12]. Mandatory notification of SBEs would identify high-risk localities and ensure or improve the supply of AVs to the relevant health centers [20]. Training of health care workers (HCWs) in the management of SBEs, starting with the selection of appropriate AVs on the basis of rational and relevant criteria, is crucial to avoid using ineffective or dangerous AVs [21]. The ASV recommended the use of effective AVs made with venoms collected from local dangerous species and validated by appropriate clinical trials [22] as preclinical neutralization tests in mice are - at best - an indication of AV efficacy in humans [21]. In addition, they must be well tolerated [i.e. preferably composed of highly purified F(ab’)_2_ that may be administered in rural peripheral health centers devoid of adverse event treatments], stable (i.e. lyophilized to avoid cold chain use that is difficult to maintain in SSA) and, if possible, affordable, even though this latter criterion is not essential for the choice of the AV [16, 17, 23].

## Strengthening Health Systems

Emergency care required to manage SBEs is very similar to many other medical conditions that need to be urgently addressed, making the integrative approach the most cost-effective. However, under the conditions currently prevailing in SSA (faulty communications, lack of transport vehicles, poor road conditions, etc.), it seems preferable to favor the provision of treatments in health centers closest to snakebite places, to shorten the delay in managing SBE, and reduce the mortality and incidence of complications, including permanent disabilities.

This was why the ASV recommended supplying AVs to peripheral health centers. In addition, the ASV is conducting prospective studies to equip these centers with broader therapeutic means, such as replacement therapy (blood transfusion, fresh frozen plasma) or manual ventilation, and train health personnel in their use. The rapid management of SBEs - and possibly other medical emergencies - in rural peripheral health centers will prevent many complications and promote the transport of patients under better conditions.

## Motivation of Communities at Risk

In addition to the beliefs of sorcery or punishment attached to snakebites that lead SSA patients to prefer traditional medicine [4, 24, 25], SBE victims avoid attending health centers that do not have adequate therapeutic means, especially AVs, as it appears valueless to them. This tradition leads to a detrimental treatment delay [26], which must be emphasized in the message intended for the communities, provided that corresponding health centers have the necessary means for effective treatment.

## Coordination of Partnerships and Resources

The global coalition advocated by the WHO roadmap [13] should include all local initiatives, adapting them to the objectives of the roadmap. In SSA, there is no financial funding for AVs, although some experiments are currently undergoing in Burkina Faso, Cameroon, Côte d’Ivoire and Togo, where local governments are supporting 50-95% of AV costs [17]. It is time for SSA governments to be involved in the considerable economic burden of SBEs and take appropriate measures funding - at least partially - the AVs. Several continental (African Union, African Development Bank, New Partnership for Africa’s Development) and regional institutions (Economic Community of West African States via the West African Health Organization, Banque de Développement des États de l’Afrique Centrale, East African Community, Southern African Development Community, etc.) can support investment and equipment, AV and drug purchase, research projects, training programs for health personnel and coordinate donations or loans from international organizations (World Bank, Islamic Development Bank, etc.) and multilateral (European Union) or bilateral cooperation. Other stakeholders should also be associated - local governments; private companies, including agricultural companies that employ a high-risk workforce; NGOs; health insurance company - should also be associated to engage in the accessibility of AV and symptomatic treatments, adjuvants or substitutes.

## A Plan for the SSA

While waiting for the development of a new generation of antivenoms based on chimeras combining representative epitopes of venom toxic antigens [27, 28], or antidotes capable of substituting or enhancing the effectiveness of AVs [29], we have to use the existing AVs, focusing on improving the manufacturing and distribution in the field.

Thus far, due to the lack of epidemiological data, financial resources and political determination, no prevention and care plan for SBEs has been proposed in SSA, either at regional or local level. Until now, local manufacturing of AVs was not considered reasonable because of the complexity and deterrent cost. Since WHO had added SBEs to its list of category A NTDs and launched its strategy for prevention and control of the SBEs, main obstacles were unlocked: the case reporting system is improving, financial resources are rising and political determination, as mentioned above, is emerging in many SSA countries [2, 17, 23].

The accessibility of the AVs can be improved by developing their manufacture inside SSA, provided the AV quality standards and production sustainability are guaranteed. The local manufacture of antivenoms is controversial because it represents considerable investments that should not lead to waste of resources [30]. In addition, it is not considered favorably by current manufacturers of antivenoms who fear harmful competition to their business. However, beyond the financial risks that need to be identified and controlled, the advantages of local antivenom manufacture should be considered. On the one hand, it will improve the perception and awareness of antivenoms by health authorities and antivenom users, both health personnel and patients. On the other hand, provided that good manufacturing practices are respected, it becomes possible to adjust the antivenom to local needs, both qualitatively (efficiency and tolerance) and quantitatively according to the incidence of SBE in the producing country and neighbors. This is why we propose that this should be undertaken carefully and progressively to ensure a positive and sustainable result.

Polyvalent AVs should be preferred because of the diversity of venomous species in SSA and difficulty of identifying the snake involved in an accident. The annual need for AVs in SSA can be estimated at more than 2 million doses, which requires several AV manufacturers to meet the demand. However, a certain number of precautions must be taken during the development of the manufacture of AVs at the different stages.

- The effectiveness of AVs depends largely on the choice of venoms used for animal immunization. Within each region and country, WHO has classified snakes into two categories: the first deals with snakes of the highest medical importance - i.e. the most common venomous species responsible for the highest morbidity and mortality -, while the second regards less venomous and/or less frequent species. It is crucial that antivenoms used in each country neutralize the venoms from all Category 1 snakes and, preferably, at least most of Category 2 snakes [31]. As a consequence, venoms should be collected from snakes originating from each area where the AVs are used. The establishment of snake farms to supply with appropriate venoms AV manufacturers would help to solve this issue. Snake farms already exist in SSA. However, most do not fulfill the standards of animal welfare, safe handling, especially during snake milking, and the conservation and traceability of venoms.
- Animals being immunized should be chosen from animals raised in SSA and, if possible, free from zoonosis transmissible to humans, e.g. donkeys, goats or camels rather than horses (they are quite difficult to breed in many SSA countries). It should be noted that camelid IgGs are better tolerated and more resistant to heat than other vertebrate IgGs [32, 33], making it a very attractive animal at least in savanna countries. Modern techniques now make it possible to use poultry, especially eggs from immunized hens, as a source of antibodies [34], although industrialization remains to be assessed and implemented.
- The safety of AVs results from a careful purification of immunoglobulin fragments (mammalian IgGs or bird IgYs) according to standards of good manufacturing practice. The conservation of the AVs is crucial in the climatic context of SSA, which can be solved by the freeze-drying of the IgG/IgY fragments precluding the need for cold chain.
- Quality control should be performed by independent laboratories to ensure the efficacy and safety of the AVs through pre-clinical tests validated by clinical trials [10, 21].

The achievement of this plan requires technology transfer for the maintenance of snake farms, venom milking and traceability, animal immunization, plasma treatment, enzymatic IgG/IgY digestion, purification and lyophilization of F(ab’)_2_. The transfer of technology will have to be compensated by an interest of the firms that will be involved, for example, in the form of profit-sharing. Governments will be responsible for finding local support from international donors or private companies. A financial contribution from the governments is essential to ensure the long-term sustainability of the local manufacture of the AVs. They can offer land without charging, pay salaries, grant subsidies, reduce taxes and, most importantly, guarantee a minimum order of AVs.

The development of AVs in SSA can be segmented by prioritizing venom sampling to supply manufacturers outside the SSA. In a second step, the immunizations could be carried out in animals raised in SSA and the plasma sent to the external manufacturers. The latter could, in a third time, deliver the F(ab’)_2_ for filling and labeling the vials in local laboratories. Finally, when each of these steps is mastered, the entire process would be performed in SSA. The time spent for the development could be used to consolidate national or regional funding, train staff, build and equip facilities, and improve AV distribution on the basis of updated epidemiological information. It will also serve to select and validate one of the alternatives between two strategies: either several local manufacturers, less expensive, more responsive and better adapted to local conditions, or a small number of regional manufacturers to better sharing resources and means.

Improving the management of SBEs in SSA will only be possible if the local populations prioritize the issue and push political and health authorities to take appropriate measures. The international community can intervene by mobilizing resources to, on the one hand, direct field research towards reasonable and affordable solutions, particularly on AVs and symptomatic, adjuvant or substitution treatments; and on the other hand support regional, national and local financial efforts. These will allow the necessary investments and supplies, as well as the training of health personnel. Supports and interventions need to be coordinated. This implies that planning is achieved through the cooperation of all stakeholders, including experts who, under the guidance of WHO, should agree on the implementation and adjustment of tactics leading to the prevention and control of snakebite envenoming according to the real needs of concerned populations.

## Abbreviations

AVs: antivenoms; HCWs: health care workers; LMICs: low and middle income tropical countries; NGOs: non-governmental organizations ; NTDs: neglected tropical diseases; SBE: snakebite envenoming; SSA: sub-Saharan Africa; WHO: World Health Organization; WHO-SBEWG: WHO Snakebite Envenoming Working Group.
